# Sealer and Moisture-Based Approach (SAMBA) Hepatectomy Technique for Robotic Parenchymal Transection

**DOI:** 10.1245/s10434-026-19372-z

**Published:** 2026-03-05

**Authors:** Emrullah Birgin, Nuh N. Rahbari

**Affiliations:** Department of General and Visceral Surgery, Ulm University Hospital, Ulm, Germany

**Keywords:** Parenchymal transection, Rrobotic liver resection, Minimally invasive hepatectomy, SynchroSeal, VesselSealer

## Abstract

**Background:**

Robotic sealing devices, such as SynchroSeal and Vessel Sealer Extend, have shown that parenchymal transection is possible without using laparoscopic instruments Finotti (Hepatobiliary Surg Nutr. 12:56–68, 2023), Palucci (J Robot Surg 19(1):36, 2024), Birgin (Lancet Reg Health Eur 43, 2024). However, there is still a need for detailed procedural guidance to ensure that these techniques can be applied effectively and consistently.

**Methods:**

We describe the Sealer and Moisture-Based Approach (SAMBA) hepatectomy technique a standardized purely robotic approach for hepatic parenchymal transection that combines robotic vessel-sealing tools with targeted saline irrigation. Between February 2021 and October 2024, a total of 72 consecutive robotic hepatectomies were performed using the SAMBA hepatectomy technique at Ulm University Hospital.

**Results:**

The Da Vinci Xi-system was used for all hepatectomies. Parenchymal transection was performed with the SynchroSeal in 55 and the Vessel Sealer Extend in 17 cases. Of these, 27 resections were nonanatomical, and 45 resections were anatomical hepatectomies with a median operative time of 174 minutes (interquartile range [IQR] 134–236), and blood loss of 200 mL (IQR 100–400). No cases of posthepatectomy hemorrhage or mortality were observed within 90 days after surgery.

**Conclusions:**

SAMBA hepatectomy technique is an effective method for robotic liver parenchymal transection. Incorporating moisture during sealing improves tissue handling, minimizes carbonization, and enhances visualization. Prospective studies are warranted to compare the SAMBA technique with other techniques of parenchymal transection.

**Supplementary Information:**

The online version contains supplementary material available at 10.1245/s10434-026-19372-z.

Minimally invasive liver surgery has evolved from laparoscopic to robotic hepatectomy, with the robotic approach providing enhanced visualization, dexterity, and precision.^1^ While laparoscopic parenchymal transection techniques have become standardized over recent decades, the range of instruments available for parenchymal transection on the robotic platform remains limited.^2^ As a result, many hepatobiliary centers continue to rely on hybrid parenchymal transection techniques. Although robotic sealing devices, such as the SynchroSeal and Vessel Sealer Extend, have demonstrated the feasibility of parenchymal transection without the need for laparoscopic instruments, comprehensive procedural descriptions detailing effective and reproducible application of these techniques are still lacking.^3^ The Sealer and Moisture-Based Approach (SAMBA) hepatectomy technique was developed to address this gap by combining robotic vessel-sealing technology with targeted saline irrigation, enabling a standardized and purely robotic approach to hepatic parenchymal transection.

## Operative Technique

**Fig. 1:**
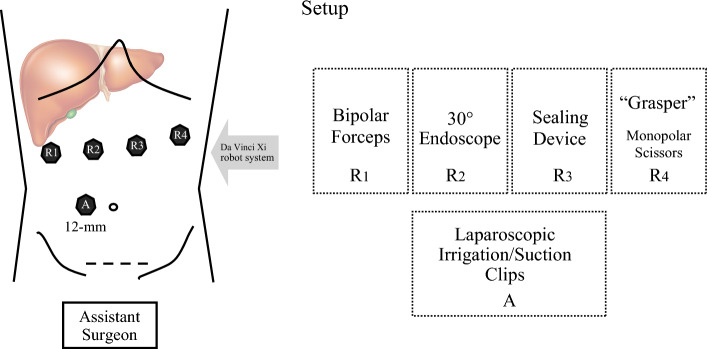
Robotic setup

**Fig. 2:**
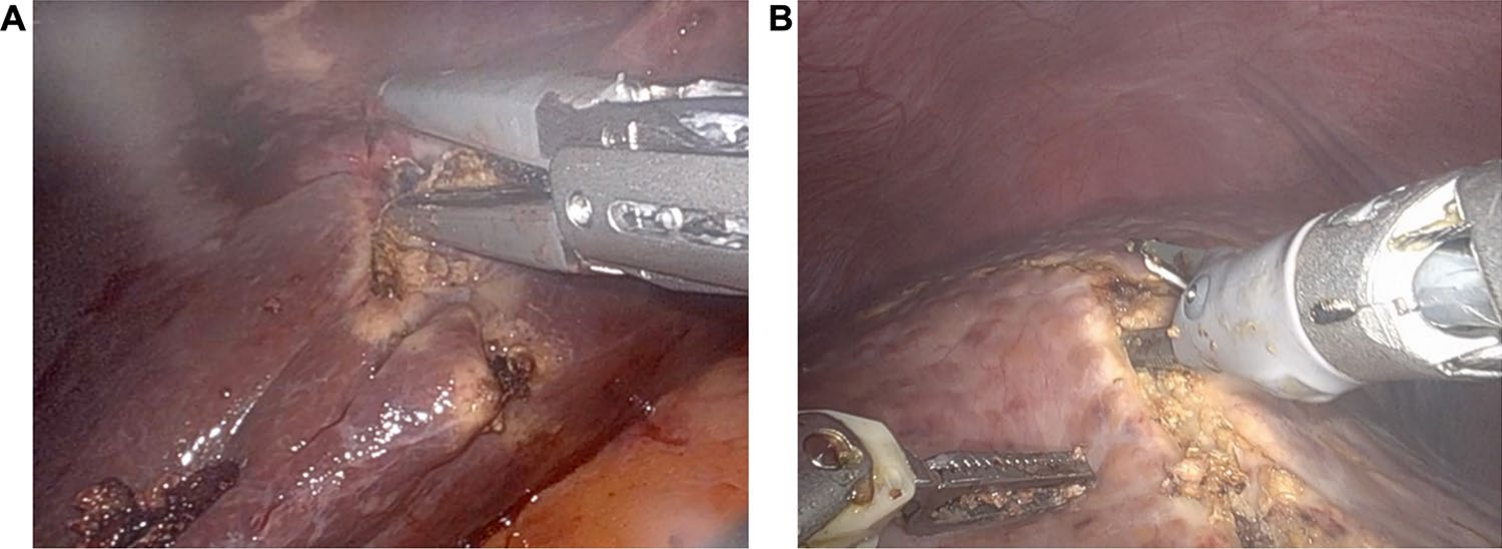
Capsule incision. Vessel Sealer Extend **A**, and SynchroSeal **B**

**Fig. 3:**
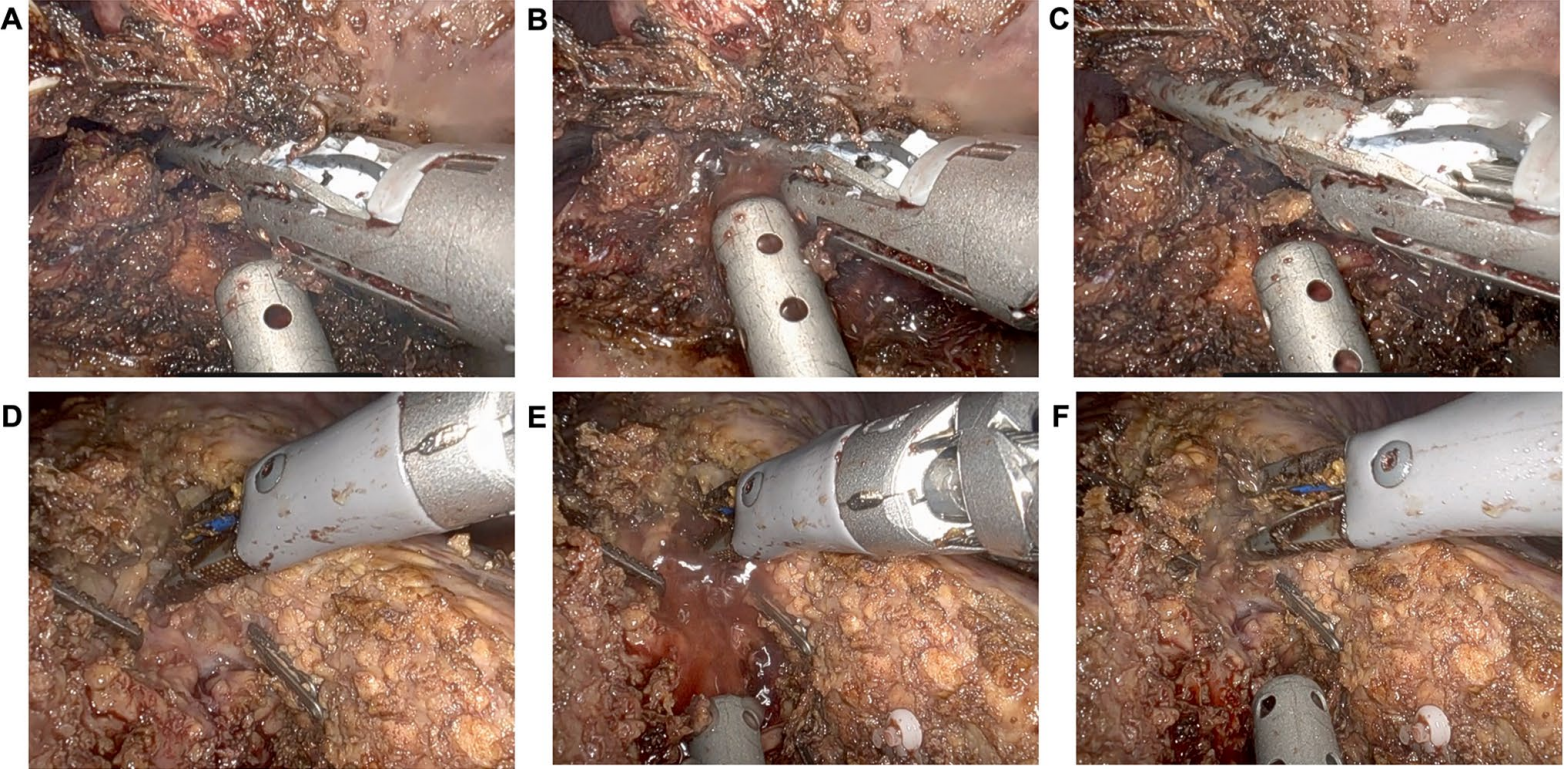
Parenchymal transection. Vessel Sealer Extend **A**–**C**, and SynchroSeal **D**–**E**

**Fig. 4:**
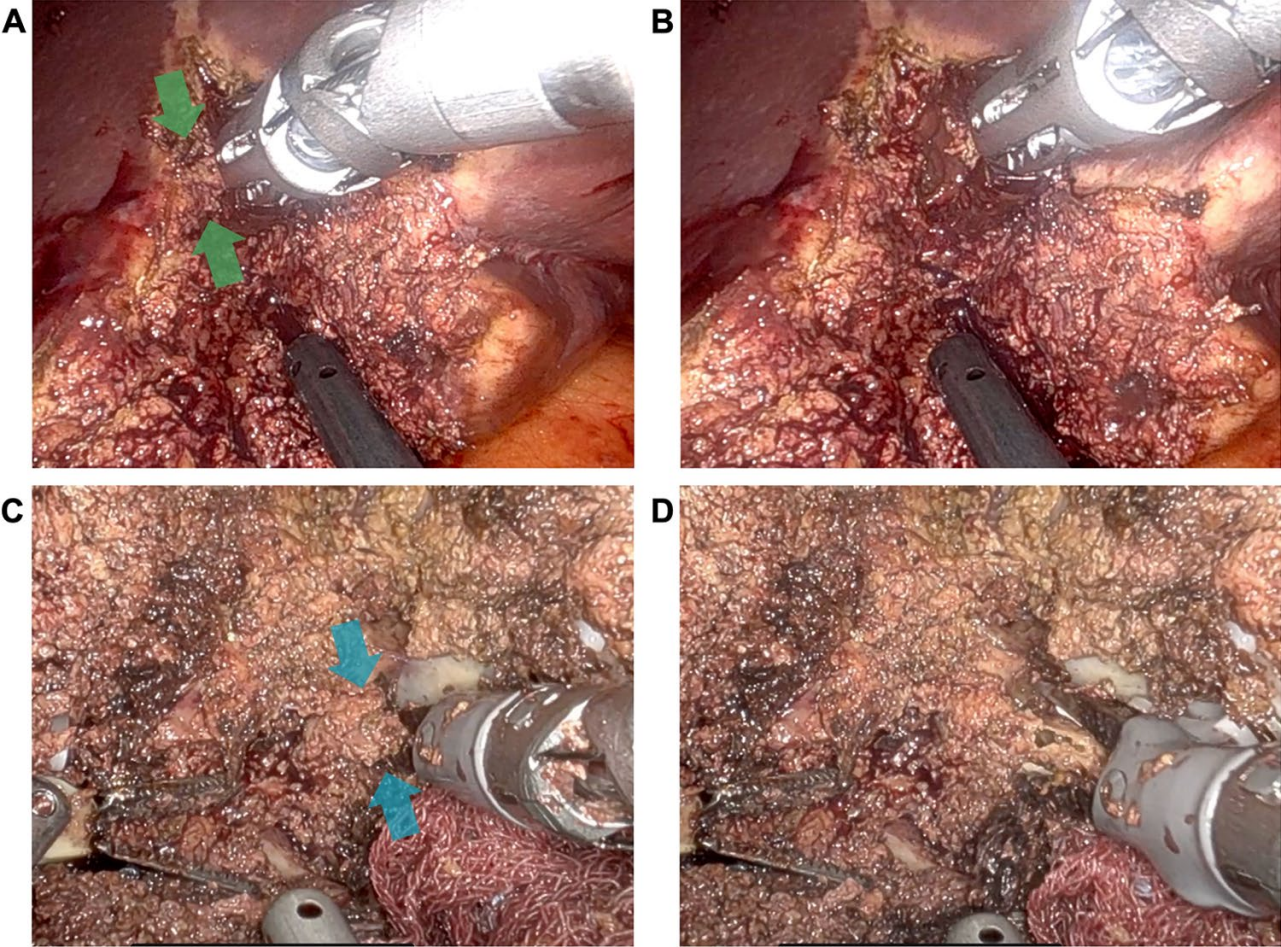
Crush-Clamping. Vessel Sealer Extend **A**–**B**, and SynchroSeal **C**–**D**

**Fig. 5:**
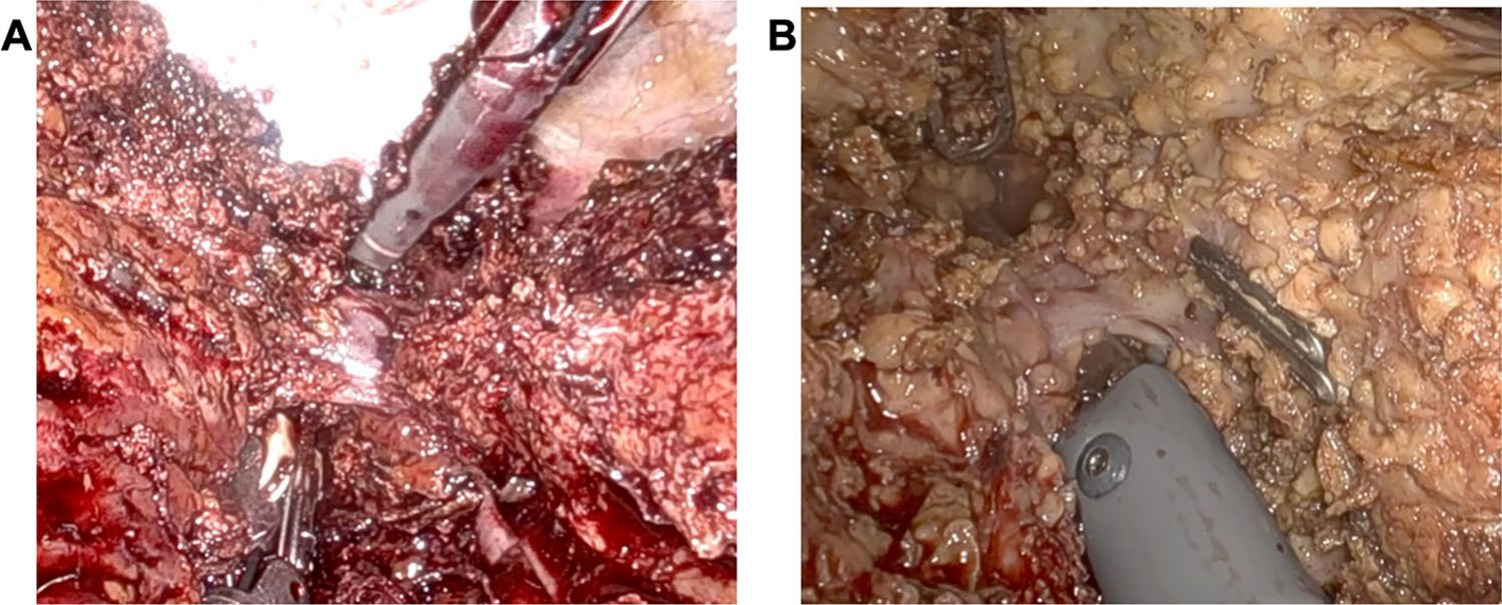
Dissection. Vessel Sealer Extend **A**, and SynchroSeal **B**

Patients are positioned supine in the split-leg and reverse Trendelenburg position. Trocar placement is illustrated in Fig. 1. Four 8-mm robotic trocars are aligned obliquely from the right lower to the left upper quadrant. The setup includes the SynchroSeal or Vessel Sealer Extend (right robotic arm), fenestrated bipolar forceps (left arm), monopolar scissors or the ProGrasp forceps (far-right arm), and a 30° endoscope. For cranio-caudal resections, such as anatomical segment 8 resections, the sealing device may alternatively be positioned in the far-right arm. A 12-mm assistant port is placed in the right lower quadrant for suction and saline irrigation. The *da Vinci Xi* system is docked from the patient’s left side. Intraoperative ultrasound is used to confirm the resection strategy and delineate the transection plane. An intracorporeal Pringle maneuver (intermittent 15–20 min occlusion with 5 min reperfusion using a Foley catheter) is applied as needed. Pneumoperitoneum is maintained at 12–15 mmHg during parenchymal transection. The liver capsule is incised using the tip of the sealing device with the jaws in a semi-open position (Fig. 2). This maneuver is facilitated by activating the "seal" or "coagulation" function (blue pedal) just before contacting the liver capsule. The SynchroSeal features sharp, curved jaws, while the Vessel Sealer Extend has straight, blunt jaws with an integrated cutting system. Consequently, incising the liver is easier with the SynchroSeal, particularly in fibrotic livers, while the cutting function of the Vessel Sealer Extend facilitates progression. Alternatively, the bipolar forceps or the monopolar scissors may be used to crush or carbonize the capsule before introducing the sealing device into the parenchyma. During superficial transection (up to 8 mm in depth), the sealing devices are used in a semi-open position, activating the “seal” and subsequently the “sync” or “cut” function. To prevent tissue adherence, the assistant intermittently irrigates the transection plane with 5–10 mL saline boluses followed by gentle suction (Fig. 3). Access to deeper parenchymal layers is facilitated by coordinated traction and countertraction using the left and far-right arm. Using approximately two-thirds of the sealing device’s jaws, a crush-clamp technique is applied to expose the underlying hepatic architecture (Fig. 4). Small vessels (≤3 mm) are divided using the “seal” and “sync” functions of the SynchroSeal or the “seal” and “cut” functions of the Vessel Sealer. Segmental pedicles or veins are managed with clips (metal or Hem-o-lok) or staplers. A precise dissection of hepatic pedicles and veins is feasible pushing the liver parenchyma with the tip of the sealing device or applying crush-clamping (Fig. 5). Large hepatic veins or segmental pedicles may be encircled with a 0-Vicryl ligature (12-cm length) before transection. Minor parenchymal oozing is managed with the bipolar forceps for localized hemostasis or, alternatively, by gently opening the sealing device’s jaws and activating the “coag” function directly on the resection plane. As parenchymal transection progresses, the frequency of irrigation is increased to minimize carbonization and prevent sticking of the sealing device. The coordinated rhythm between the surgeon and assistant—alternating sealing, irrigation, and release—enhances the precision of the seal-divide release sequence and ensures steady progress through the parenchymal plane. Laparoscopic gauzes are used intracorporeally with irrigation to clean the jaws of the sealing device with the bipolar forceps if significant carbonization occurs, avoiding the need for extracorporeal cleaning. After completing the resection, pneumoperitoneum is reduced to 10 mmHg to inspect for bleeding or bile leakage. The specimen is retrieved in a bag via a Pfannenstiel incision, without routine placement of abdominal drains.

## Results

Between February 2021 and October 2024, a total of 72 consecutive robotic hepatectomies were performed using the SAMBA hepatectomy technique at Ulm University Hospital. Of these, the SynchroSeal was used in 55 and the Vessel Sealer Extend in 17 cases. Twenty-seven resections (38%) were nonanatomical, while 45 (63%) were anatomical hepatectomies. The median age was 64 years (interquartile range [IQR] 54–68), and 52% were male. Indications for surgery included liver metastases (n = 24), hepatocellular carcinoma (n = 11), cholangiocarcinoma (n = 5), and benign lesions (n = 32). Nine patients had underlying liver cirrhosis. The median operative time was 174 minutes (IQR 134–236), and the median estimated blood loss was 200 mL (IQR 100–400). Thirteen patients experienced intraoperative blood loss exceeding 500 mL. Four patients (6%) developed Grade B bile leaks, all of which were managed conservatively with percutaneous drainage and endoscopic biliary stent placement. No cases of posthepatectomy hemorrhage or mortality were observed within 90 days after surgery**.**

## Conclusions

SAMBA hepatectomy technique is an effective method for robotic liver parenchymal transection. Incorporating moisture during sealing improves tissue handling, minimizes carbonization, and enhances visualization, particularly in deeper parenchymal layers. Prospective studies are warranted to compare the SAMBA technique with other techniques of parenchymal transection in terms of operative efficiency, safety, and long-term outcomes.

## Supplementary Information

Below is the link to the electronic supplementary material.

Supplementary file1 (MP4 231332 KB)

## References

[1] Finotti M, D’Amico F, Mulligan D, Testa G. A narrative review of the current and future role of robotic surgery in liver surgery and transplantation. *Hepatobiliary Surg Nutr*. 2023;12(1):56–68. 36860258 10.21037/hbsn-21-115 PMC9944521

[2] Palucci M, Giannone F, Del Angel-Millan G, et al. Robotic liver parenchymal transection techniques: a comprehensive overview and classification. *J Robot Surg*. 2024;19(1):36. 39738738 10.1007/s11701-024-02200-5

[3] Birgin E, Heibel M, Hetjens S, et al. Robotic versus laparoscopic hepatectomy for liver malignancies (ROC’N’ROLL): a single-centre, randomised, controlled, single-blinded clinical trial. *Lancet Reg Health Eur*. 2024;43:100972. 39210947 10.1016/j.lanepe.2024.100972 PMC11360176

